# First detection, isolation and molecular characterization of infectious salmon anaemia virus associated with clinical disease in farmed Atlantic salmon (*Salmo salar*) in Chile

**DOI:** 10.1186/1746-6148-4-28

**Published:** 2008-08-04

**Authors:** Marcos G Godoy, Alejandra Aedo, Molly JT Kibenge, David B Groman, Carmencita V Yason, Horts Grothusen, Angelica Lisperguer, Marlene Calbucura, Fernando Avendaño, Marcelo Imilán, Miguel Jarpa, Frederick SB Kibenge

**Affiliations:** 1 Biovac SA, Puerto Montt, Chile; 2 Department of Pathology and Microbiology, OIE Reference Laboratory for ISA, Atlantic Veterinary College, University of Prince Edward Island, Charlottetown, PE, Canada; 3 Aquatic Diagnostic Services, Atlantic Veterinary College, University of Prince Edward Island, Charlottetown, PE, Canada; 4 Regional Diagnostic Virology Laboratory, Atlantic Veterinary College, University of Prince Edward Island, Charlottetown, PE, Canada; 5 Marine Harvest S.A., Puerto Montt, Chile

## Abstract

**Background:**

Infectious salmon anaemia (ISA) is a viral disease of marine-farmed Atlantic salmon (*Salmo salar*) caused by ISA virus (ISAV), which belongs to the genus *Isavirus*, family *Orthomyxoviridae*. The virus is considered to be carried by marine wild fish and for over 25 years has caused major disease outbreaks in marine-farmed Atlantic salmon in the Northern hemisphere. In the Southern hemisphere, ISAV was first detected in Chile in 1999 in marine-farmed Coho salmon (*Oncorhynchus kisutch*). In contrast to the classical presentation of ISA in Atlantic salmon, the presence of ISAV in Chile until now has only been associated with a clinical condition called Icterus Syndrome in Coho salmon and virus isolation has not always been possible. During the winter of 2007, unexplained mortalities were registered in market-size Atlantic salmon in a grow-out site located in Chiloé in Region X of Chile. We report here the diagnostic findings of the first significant clinical outbreak of ISA in marine-farmed Atlantic salmon in Chile and the first characterization of the ISAV isolated from the affected fish.

**Results:**

In mid-June 2007, an Atlantic salmon marine farm site located in central Chiloé Island in Region X of Chile registered a sudden increase in mortality following recovery from an outbreak of Pisciricketsiosis, which rose to a cumulative mortality of 13.6% by harvest time. Based on the clinical signs and lesions in the affected fish, and laboratory tests performed on the fish tissues, a confirmatory diagnosis of ISA was made; the first time ISA in its classical presentation and for the first time affecting farmed Atlantic salmon in Chile. Rapid sequencing of the virus-specific RT-PCR products amplified from the fish tissues identified the virus to belong to the European genotype (Genotype I) of the highly polymorphic region (HPR) group HPR 7b, but with an 11-amino acid insert in the fusion glycoprotein, and ability to cause cytopathic effects (CPE) in CHSE-214 cell line, characteristics which make it distinct from common European Genotype ISAV isolates from Europe and North America.

**Conclusion:**

In conclusion, the present work constitutes the first report of a case of ISA in farmed Atlantic salmon in Chile. The clinical signs and lesions are consistent with the classical descriptions of the disease in marine-farmed Atlantic salmon in the Northern hemisphere. The outbreak was caused by ISAV of European genotype (or Genotype I) of HPR 7b but distinct from common European Genotype ISAV isolates.

## Background

Infectious salmon anaemia (ISA) is a viral disease of marine-farmed Atlantic salmon (*Salmo salar*) caused by ISA virus (ISAV), which belongs to the genus *Isavirus*, family *Orthomyxoviridae *[1]. In the Northern hemisphere, the first registered outbreak of ISA was in 1984 in Atlantic salmon "parr", on the southwestern coast of Norway [2]. Subsequently the disease was reported in Canada in 1996 [3], in Scotland in 1998 [4], in Faeroe Islands in 1999 [5], and in Maine, USA, in 2000 [6]. The clinical disease in farmed Atlantic salmon is characterized by variable mortality ranging from 0 to 50% with ascites, exophthalmia, petechiation of the visceral adipose tissue, haemorrhagic liver necrosis, renal interstitial haemorrhage and tubular nephrosis, filamental sinus congestion of the gills, splenic congestion with concomitant erythrophagocytosis, and congestion of the lamina propria of the stomach and foregut [2,7-10]. ISAV remains an emerging fish pathogen that continues to cause severe economic losses to the salmon-farming industry in an increasing number of countries, although the disease can be successfully eradicated as was managed by Scotland in the late 1990s [11]. In the Southern hemisphere, ISAV was first detected in Chile in 1999 in marine-farmed Coho salmon (*Oncorhynchus kisutch*) and was shown to be of the North American genotype [12]. In contrast to the classical presentation of ISA in Atlantic salmon, the presence of ISAV in Chile up until now has only been associated with a clinical condition called Icterus Syndrome in Coho salmon and virus isolation has not always been possible [13]. Between 2001 and 2003, ISAV of the North American genotype was found in apparently normal Atlantic salmon in Lake Llanquihue (Oscar Gárate, personal communication), which is not in the same zone as where the present disease outbreak occurred. It is not known why the North American variant of ISAV has not caused typical ISA outbreaks as those known to occur in Eastern Canada and Maine, USA, and yet the virus has become widespread in the Atlantic salmon industry in Chile.

ISAV belongs to the family *Orthomyxoviridae*, together with influenza viruses [1]. However, the virus is sufficiently different from influenza viruses to be assigned to its own genus, *Isavirus*. Members of this genus are enveloped particles of 90–140 nm diameter with surface projections consisting of a combined haemagglutinin-esterase (HE) glycoprotein, which is the receptor binding haemagglutinin with receptor destroying enzyme activity demonstrated to be an acetylesterase [14,15] encoded on segment 6 [14], and a separate fusion (F) glycoprotein encoded on segment 5 [16]. The genome consists of eight segments of linear, single-stranded negative sense RNA ranging in length from 1.0 to 2.4 kb with a total molecular size of approximately 14.3 kb [17]. Sequence analysis of several ISAV isolates on the eight segments consistently reveals two genotypes that are designated with respect to their geographic origin, European and North American, hereafter designated Genotypes I and II, respectively. Sequence variation in a 35-amino acid highly polymorphic region (HPR) on the HE glycoprotein stalk has allowed the separation of ISAV isolates into different HPR groups, with the HPR0 group consisting of the non-cultivable, non-pathogenic viruses detectable only by RT-PCR [18] whereas deletion in the HPR of ≥ 13 amino acids (or if less, with deletion or mutation of the motif at amino acid positions ^352^FNT^354^) lead to increased pathogenicity, and ability to replicate in cell culture with production of CPE and consequent virus isolation [19].

This study reports the diagnostic findings of the first occurrence of ISA in the Southern hemisphere, and the first characterization the ISAV isolated from the affected marine-farmed Atlantic salmon in Chile.

## Results

### Signalment

**Figure 1:**
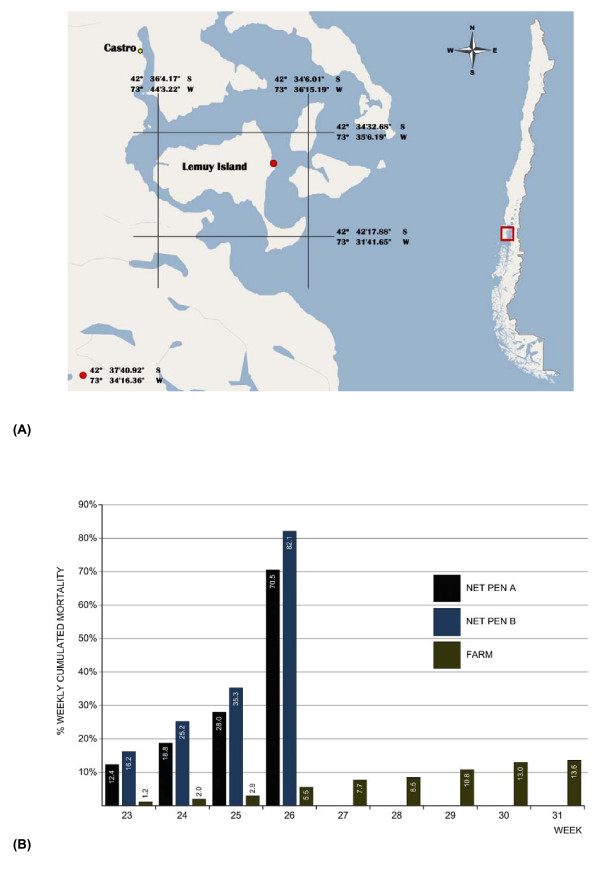
**Geographical location and mortality pattern of the first ISA outbreak in Chile**. (A)Geographical location of the Atlantic salmon marine farm site: Lemuy Island, Central Chiloé, Region X, Chile. (B)Weekly percent cummulative mortality up to the time of total harvest of the Atlantic salmon marine farm with ISA outbreak. Net Pen A and Net Pen B represent mortality in the 2 cages with ISA. FARM corresponds to the total mortality on the farm up to the time of harvest.

In mid-June 2007 following recovery from an outbreak of Pisciricketsiosis, a severe increase in mortality was recorded in 2 cages on an Atlantic salmon grow-out site located in central Chiloé in Region X of Chile (Fig. 1A) [see Additional file 1]. The moribund fish were lethargic and had dark integument. By the time the fish were removed from the cages 4 weeks later, the cumulative mortality had reached 70% in one cage and 82% in the second cage. Concomitantly, in other sites of the same area, high mortalities attributed to amoebas and flexibacteriosis were reported, which added to the diagnostic confusion. The total cumulative mortality on the index farm was only 13.6% at the time of harvest due to company decision to cull all remaining cages independently of their clinical status (Fig. 1B).

### Gross pathology

**Figure 2:**
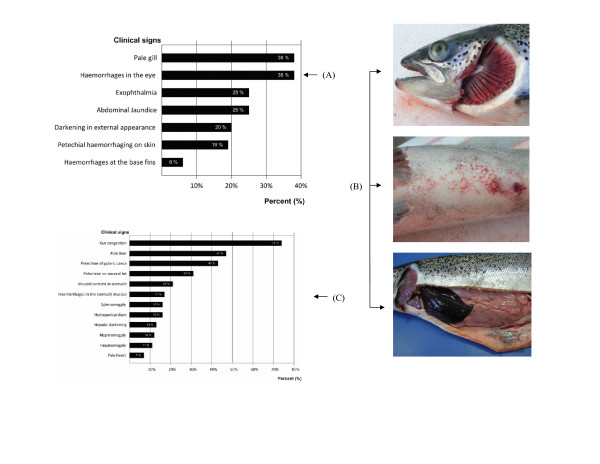
**Gross lesions in affected Atlantic salmon from the ISA outbreak**. (A) Frequency of the external gross lesions in affected Atlantic salmon from the ISA outbreak. Percentage of fish with a specified lesion among 100 fish necropsied. (B) Common gross lesions seen at necropsy: Top panel – Atlantic salmon with exophthalmia and pale gills. Middle panel – Atlantic salmon with petechial haemorrhages on the abdomen. Bottom panel – Atlantic salmon with very dark liver and haemorrhages on the visceral adipose tissue. (C) Frequency of the internal gross lesions in affected Atlantic salmon from the ISA outbreak. Percentage of fish with a specified lesion among 100 fish necropsied.

The external gross findings noted on Atlantic salmon that were necropsied included exophthalmia, periocular haemorrhage and darkening of the integument. Figure 2A summarizes the frequency of the external lesions noted from 100 affected fish at necropsy. Periocular haemorrhage and paleness of the gills were observed in 38% of the fish, whereas 25% presented with exophthalmia [see Additional file 2] and jaundice in the ventral zone (Fig 2B). The presence of haemorrhages in the central zone was observed in 19% of the fish (Fig. 2B). Of the 100 fish that were examined, 63% were also infested with *Caligus sp*. At necropsy, the internal gross findings included haemorrhaging in the visceral adipose tissue, liver, stomach and gut, with the liver and spleen of some fish appearing dark (Fig. 2B). Some fish had hydropericardium [see Additional file 3], and enlarged spleen and kidney. Figure 2C summarizes the frequency of the internal gross lesions noted from 100 affected fish which were necropsied in this outbreak. The most common lesions were haemorrhagic enteritis in 64%, haemorrhages in pyloric caeca in 43%, and haemorrhages in visceral adipose tissue in 31% of the cases [see Additional files 4 and 5].

### Histopathology

**Figure 3:**
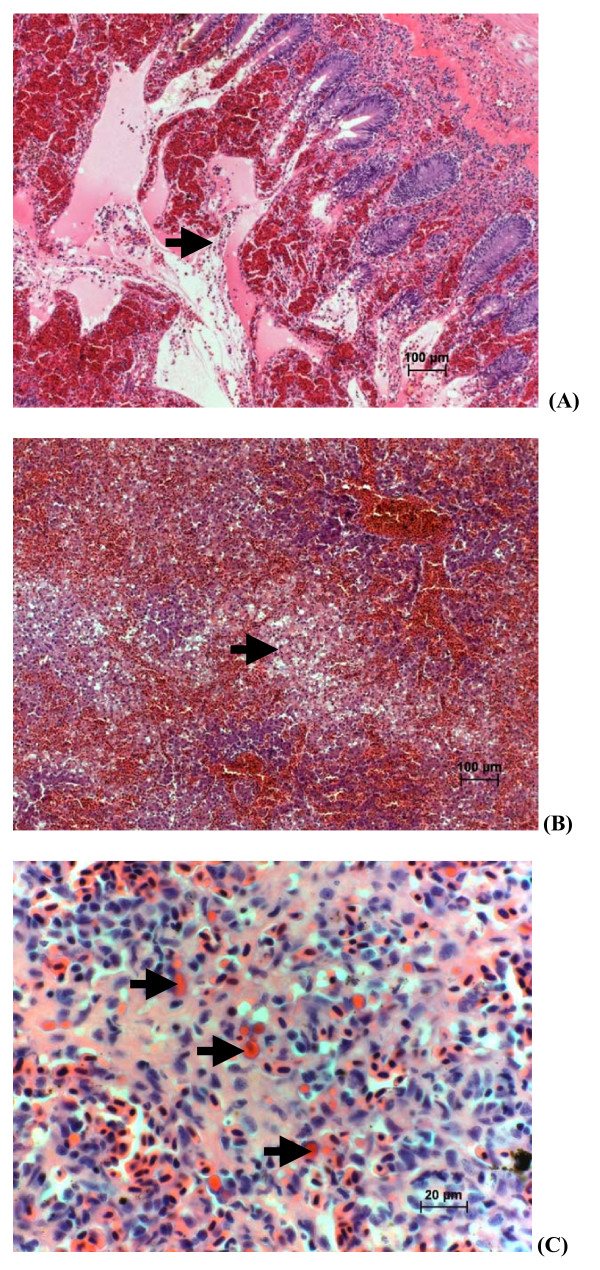
**Microscopic lesions in affected Atlantic salmon from the ISA outbreak**. (A) Histologic section of intestine of Atlantic salmon. H&E staining. (→) indicates mucosal ulceration and haemorrhage. (B) Histologic section of liver of Atlantic salmon from the ISA outbreak H&E staining. (→) indicates hepatocellular necrosis, with marked haemorrhage and sinusoidal congestion/peliosis. (C) Histologic section of spleen of Atlantic salmon from the ISA outbreak H&E staining. (→) indicates endothelial erythrophagia.

Diffuse congestion of lamellar capillaries, with marked infiltration of the filamental subcutis by eosinophillic granular leucocytes was evident in gill tissue. The kidney showed mild diffuse sinusoidal congestion with evidence of increase in circulating granulocytes and intravascular erythrophagia. The intestine and pyloric caeca were the most severely affected, showing marked congestion of the lamina propria vasculature with an associated mixed leucocytic infiltration of submucosa and localized perivascular and intra-lumenal haemorrhage (Fig. 3A). The liver was affected by multifocal to coalescing regions of sinusoidal congestion and peliosis, with mild adjacent hepatocellular necrosis (Fig. 3B). Changes in the spleen included marked sinusoidal congestion and intravascular erythrophagia (Fig. 3C). Mucosal epithelium in the most severely affected regions was denuded. There were no significant morphologic changes noted in the heart, stomach or body wall skeletal muscle. Morphologic diagnoses included: a) necrotizing and congestive hepatitis, subacute, multifocal to bridging, moderate to marked; b) ulcerative mixed leucocytic and congestive enteritis, with marked intra-lumenal haemorrhage, subacute, moderate to marked; and d) congestive splenitis and interstitial nephritis, with intravascular erythrophagia, moderate to marked.

### Virus antigen detection

**Figure 4:**
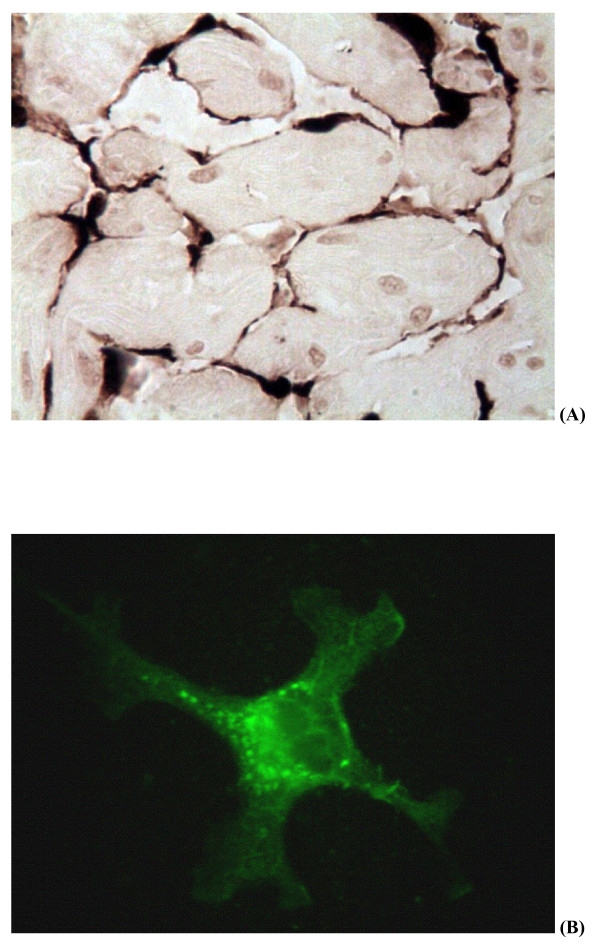
**ISAV antigen staining of samples from the ISA outbreak**. (A) Immunohistochemical staining of histologic section of heart of Atlantic salmon from the ISA outbreak. Dark brown colour indicates positive staining in endothelial cells with anti-ISAV monoclonal antibody. (B)Virus isolation in CHSE-214 cells: ISAV-infected cells (250×) showing fluorescent staining following IFAT with anti-ISAV monoclonal antibody.

Sections of the kidney, spleen, heart, gills and intestines were subjected to immunohistochemistry staining with anti-ISAV monoclonal antibody P10 (Aquatic Diagnostics Ltd, Stirling, Scotland). ISAV proteins were detected in all the organs tested. Figure 4A shows positive staining of endothelial cells of the heart. Homogenates of pooled spleen and kidney tissues used for virus isolation were also tested in a rapid kit made by Aquatic Diagnostics, based on immunochromatography with anti-ISAV monoclonal antibody. The samples taken from affected fish were positive for ISAV. The infectious haematopoietic necrosis virus (IHNV) ELISA kit and viral haemorrhagic septicaemia virus (VHSV) ELISA kit (Bio-X Diagnostics, Jemelle, Belgium) were used to rule out the presence of IHNV and VHSV in the tissue samples.

### Virus isolation

Homogenates of pooled spleen and kidney tissues inoculated on Chinook salmon embryo (CHSE-214), Salmon head kidney (SHK-1) and *Epithelioma papulosum cyprini *(EPC) cell lines produced CPE between 4 and 7 days post inoculation during primary isolation. The CPE, which was reproduced on subsequent passage on fresh cell line monolayers, was characterized initially by cell vacuolization and rounding and then detachment from the substrate involving the whole cell monolayer. The presence of ISAV in the cell lysates was confirmed using RT-PCR and an indirect immunofluorescent antibody test (IFAT) with anti-ISAV monoclonal antibody P10 (Aquatic Diagnostics Ltd) including appropriate controls. Figure 4B shows positive IFAT of a CHSE-214 cell culture inoculated with a tissue sample from affected fish. Similar fluorescence was seen with EPC cell cultures with CPE (data not shown). Neither CPE nor presence of ISAV was detected by RT-PCR or IFAT in inoculated cultures of Bluegill Fry (BF2) cell line.

### RT-PCR

Tissue samples shipped in RNA *later*^® ^(Ambion Inc., Foster City CA) to the Regional Diagnostic Virology Laboratory, AVC, UPEI, Canada, were positive for ISAV by RT-PCR and negative for both IHNV and VHSV by nested RT-PCR.

### Sequence analysis

The nucleotide sequences in this report, which are all of ISAV RNA segment 5, are available through GenBank , accession nos. EU130923, EU486160, and EU486161. A partial sequence of ISAV RNA segment 6 was generated by the Norwegian Veterinary Institute from duplicate samples of the same ISA outbreak, and appears in GenBank, accession no. AM941715. Tables 1 [see Additional file 6] and 2 [see Additional file 7] show the percent sequence identities between the new Chile ISAV and selected ISAV isolates from Europe and North America. Comparison of the RNA segment 5 sequences of the Chilean ISAV with ISAV strains of Genotype I (European genotype) showed nucleotide sequence identities ≥ 95.9% whereas with ISAV strains of Genotype II (North American genotype), it was ≤ 74.7% [see Additional file 6]. Comparison of the segment 6 sequence of the Chilean ISAV with ISAV strains of Genotype I showed sequence identities ≥ 95.7% whereas with ISAV strains of Genotype II, it was ≤ 79.4% [see Additional file 7]. Thus the new Chile ISAV involved in this outbreak belongs to Genotype I (the European genotype).

**Figure 5:**
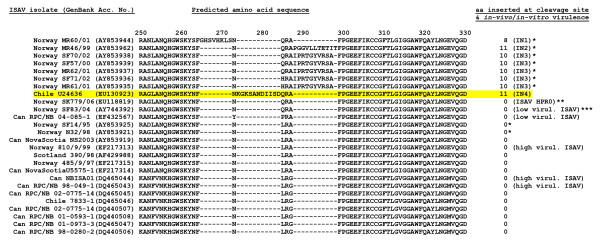
**Alignment of amino acid sequences in the proteolytic cleavage site of the precursor F_0 _protein (modified from Kibenge *et al***. [19]**).** The amino acid sequence corresponding to the fusion protein of the Chilean ISAV in this disease outbreak is highlighted in yellow. The designation of amino acid inserts IN1, IN2, and IN3 are as reported by Devold *et al*. [21]. The unique 11-amino acid insert found in the Chilean ISAV in this disease outbreak is designated IN4. Other sources of information are indicated as *Devold *et al*. [21], **Markussen *et al*. [20], and ***Plarre and Nylund (2004; SF83/04, GenBank Accession No. AY744392). It has been suggested by Markussen *et al*. [20] that ISAV isolate SF83/04 represents a mixed virus infection of HPR0 and another HPR group, which might explain the confusion in the virulence designation.

**Figure 6:**
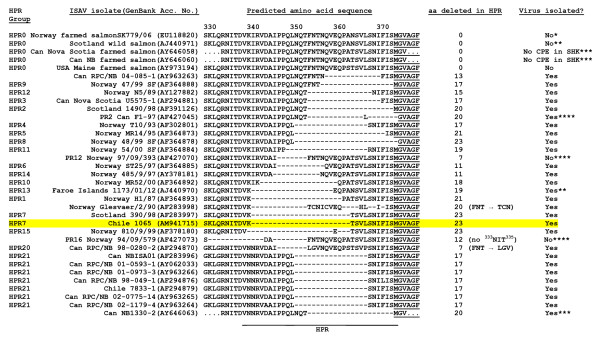
**Alignment of amino acid sequences in the highly polymorphic region (HPR) of the HE genes of various strains of ISAV (modified from Kibenge *et al***. [19]**).** The amino acid sequence corresponding to the HE-HPR of the Chilean ISAV in this disease outbreak is highlighted in yellow, and identifies it as HPR7. Sequences that are not determined are indicated by dots, and amino acid (aa) deletions in the HPR are indicated by dashes. The HPR groups identified are as reported by Nylund *et al*. [23] and Plarre *et al*. [24]; other sources of information are indicated as *Markussen *et al*. [20], **Cunningham *et al*. [25], ***Cook-Versloot *et al*. [26], and ****Mjaaland *et al*. [27].

Alignment of the amino acid sequences of RNA segment 5, which encodes the F glycoprotein, revealed that the new Chile ISAV has a small insert of 11 amino acids (or 33 nucleotides) relative to ISAV strains from Europe and North America near the putative proteolytic cleavage site of the precursor F_0 _protein (Fig. 5). This insert has 100% sequence identity with RNA segment 2 of Genotype I. ISAV RNA segment 2 encodes the PB1 polymerase [22]. Alignment of the amino acid sequences of RNA segment 6, which encodes the HE glycoprotein, revealed that the new Chile ISAV belongs to HPR7b of the European genotype as described by Nylund *et al*. [23] and Plarre *et al*. [24] (Fig. 6) and reviewed by Rimstad *et al*. [18].

## Discussion

The present work constitutes the first report of a case of ISA in farmed Atlantic salmon in Chile. The outbreak was caused by ISAV of Genotype I (the European genotype). The gross and microscopic lesions observed in this outbreak are consistent with the classical ISA described in Norway [2], Canada [3], Scotland [4], Faeroe Islands [5] and United States [6]. In particular, the liver congestion and necrosis, as well as the renal, splenic and enteric congestion/haemorrhage in this outbreak are morphologically consistent with lesions pathognomonic for ISA. However, where these lesions differed from other presentations of the disease were primarily in the kidney, where in the Canadian situation there is profound interstitial haemorrhage, and in the heart, in the Chilean ISA outbreaks there is hydropericardium and severe myocarditis. Previously, such heart lesions have been shown to be prominent only in experimental infections of rainbow trout with ISAV [28].

The affected fish group in the Chilean ISA outbreak was recovering from Piscirickettsiosis. This particular group had been treated several times against Rickettsia at the farm site, and on this occasion the antibiotic treatment which was administered by injection effectively prevented mortality due to Piscirickettsiosis. Although the pathologic findings in this outbreak were in the range of what is considered as typical ISA, it cannot be categorically stated what contribution, if any, the Piscirickettsiosis disease contributed to the pathology observed.

The virus responsible for the outbreak caused CPE and was recovered using CHSE-214, SHK-1 and EPC cell lines, and confirmed as ISAV by means of RT-PCR and IFA testing. This is the first report of primary isolation of ISAV using EPC cell line, which is a non-salmonid cell line derived from a skin tumour of carp (*Cyprinus carpio *L.) [29] and never before reported to be permissive to ISAV. Most recently, EPC cell line was also reported to be permissive to ISAV of North American genotype [30]. Moreover rapid sequencing of virus-specific RT-PCR products amplified from the fish tissues identified the virus as belonging to the European genotype. Up until now, only some strains of ISAV of the North American genotype [31-33] but not the European genotype [32] have been known to replicate and cause CPE in the CHSE-214 cell line, a property that is not associated with the antigenic subtype of ISAV nor with the HPR group of the HE gene [34]. Thus, the Chilean ISAV has cell line specificities that are different from those of common European Genotype ISAV isolates from Europe and North America.

Now that we have ISAV isolates of both genotypes from Europe, North America, and South America, we should remove the stigmatizing labeling of ISAV genotypes by geographic reference. We therefore propose to designate the European genotype as Genotype I and the North American genotype as Genotype II. Such designation is consistent with our present knowledge, which is not sufficient to explain the anomalous geographic distribution of the two genotypes of ISAV.

The Chilean ISAV responsible for the ISA outbreak is unique in that it has a 33-nucleotide (or 11-amino acid) insert in the middle of RNA segment 5 which encodes the F glycoprotein. This insert has 100% sequence identity with RNA segment 2 of ISAV of Genotype I, and probably arose through a non-homologous recombination between the F and PB1 genes of the same virus. To date, there have been only 8 other ISAV isolates with inserts in RNA segment 5 [20,21]. All these isolates are Norwegian ISAV isolates; seven of them were recovered between 1999 and 2002 [21] and one was recovered in 2006 [20]. Seven of these isolates had inserts from different parts of RNA segment 5 while in one isolate, the insert was shown to come from RNA segment 3, which encodes the ISAV nucleoprotein. Therefore, this Chilean ISAV is the first known isolate to have an insert in RNA segment 5 coming from RNA segment 2. Overall, this information indicates that this Chilean ISAV is different from common Genotype I ISAV isolates in Europe. The location of the insert in the Chilean ISAV RNA segment 5 is also unique in that it occurs right in the middle of the ^265^YP^266 ^motif, which was recently shown to be a marker for reduced virulence [19]. The RNA segment 5 of ISAV belonging to HPR0, a non-pathogenic virus, has ^265^NQ^266 ^at this site but without a sequence insertion [20], which would be similar to the Chilean ISAV prior to the non-homologous recombination event between the F and PB1 genes. Such recombination events are well known in avian influenza virus (AIV), involving an insertion in the haemagglutinin (HA) gene of AIV near the cleavage site of the protein and leading to emergence of new virulent strains [35-38]. For example, the avian influenza disease outbreak that occurred in Chile in 2002 was attributed to the non-homologous recombination between the HA and nucleoprotein genes of H7N3 AIV [38].

The Chilean ISAV described here clearly differs from the 7833-1 isolate (of Genotype II) from coho salmon in Chile [12] in segment 5 and the partial segment 6 amino acid sequences. Thus the 7833-1 virus, which is now wide spread in the Atlantic salmon industry in Chile, is unlikely to have been the source of this new virus. It is interesting that the alignments on RNA segment 6 showed the Chilean ISAV to belong to HPR7b that has previously been found in Scotland and Norway. However, all ISAV isolates of HPR7b sequenced to date on RNA segment 5 do not have the 11-amino acid insert found in the Chilean ISAV. Comparison of the RNA segment 5 sequence of this Chilean ISAV with the HPR0 virus strain SK779/06 [20] showed a nucleotide sequence identity of 98.3% and amino acid sequence identity of 97.1%. In contrast, ISAV strain 390/98, which is also of HPR7 but from Scotland, when compared to the HPR0 virus showed a nucleotide sequence identity of 98.4% and amino acid sequence identity of 99.1% [see Additional file 6]. This suggests that the Chilean ISAV is distinct from common Genotype I ISAV isolates from Europe and North America. The location of the 11-amino acid insert at a motif in the F glycoprotein which is associated with virus virulence, may account for the elusive nature of this Chilean virus. It is therefore necessary to sequence the whole genome of this virus and clarify the epidemiology of this ISA outbreak so as to establish the origin of this virus, its virulence characteristics and the risk factors associated with its presentation and dissemination, in order to institute adequate strategies for the control and prevention of ISA in the Chilean salmon industry.

## Conclusion

In conclusion, the present work constitutes the first report of a case of ISA in farmed Atlantic salmon in Chile. The clinical signs and lesions are consistent with the classical descriptions of the disease in marine-farmed Atlantic salmon in the Northern hemisphere. The outbreak was caused by ISAV of Genotype I of HPR 7b but distinct from common European ISAV isolates from Europe and North America.

## Methods

### Field sampling

Atlantic salmon with mean weight of 3.9 Kg, held in seawater rearing cages, were necropsied and submitted for diagnostic testing. Moribund fish were submitted for laboratory analysis to the Biovac S.A. laboratory in Puerto Montt, Chile, where a full necropsy was conducted and samples were collected for histological evaluation, virus isolation, and immunohistochemistry and molecular biology analysis.

### Histology

Tissue samples for histological analysis were collected in 10% buffered formalin. They were then processed using standard procedures and the sections were stained with Haematoxylin & Eosin (H&E), in order to describe the significant morphological changes. Tissues from 9 fish were additionally submitted to Aquatic Diagnostic Services at the Atlantic Veterinary College in Prince Edward Island, Canada, for histological evaluation.

### Virus antigen detection assays

The presence of virus antigens in the fish tissues utilized three different types of assays: immunohistochemistry, immunochromatography, and antigen ELISA. The immunohistochemistry analyses were carried out on waterproofed tissue sections of 5 μm thickness, using anti-ISAV monoclonal antibody P10 (Aquatic Diagnostics Ltd, Stirling, Scotland) with the immunohistochemistry kit from Vector Laboratories. Immunochromatography utilized tissue homogenates applied to a rapid kit with anti-ISAV monoclonal antibody according to the manufacturer's procedures (Aquatic Diagnostics Ltd). The IHNV ELISA kit (Bio-X Diagnostics) and VHSV ELISA kit (Bio-X Diagnostics) were used to rule out IHNV and VHSV in the tissue samples. The ELISA procedures followed the manufacturer's protocols.

### Virus isolation

Homogenized pools of spleen and kidney were inoculated on monolayers of CHSE-214, EPC, SHK-1 and BF2 cell lines following standard protocols in the OIE Aquatic Manual [39].

### RT-PCR and nucleic acid sequencing

Total RNA was extracted from the same homogenates of pooled spleen and kidney tissues used for virus isolation, using EZNA^® ^kits (Omega Biotech) or the RNeasy^® ^kit (Qiagen) according to the manufacturer's recommended protocol. For tissues preserved in RNA *later*^® ^(Ambion Inc), the tissues were first washed three times with phosphate buffered saline (PBS) and then homogenized using a tissue homogenizer (Brinkman, Ontario, Canada) prior to total RNA extraction using the RNeasy^® ^kit (Qiagen).

For the detection of ISAV in tissue homogenates, ISAV-specific primers and conditions described by Devold *et al*. [40] and Mjaaland *et al*. [41] for RNA segment 8, and the primers described in the OIE Aquatic Manual [39] for RNA segment 6 were used. Briefly, amplification was performed using 50 μl reaction mixture utilizing EzrTth RT-PCR kit (Applied Biosystems, Montreal, Quebec) as follows: EzrTth DNA polymerase – 1 μl, ISA forward primer-0.4 uM, ISA reverse primer- 0.4 uM, Mn-2.5 mM, dNTPs-200 uM each, 5× PCR buffer-10 μl, ddH_2_O-19 μl and 5 μl RNA template. The amplification was performed in Perkin Elmer Gene Amp PCR System 2400 using the following conditions: 1 cycle at 53°C for 45 min, one cycle at 94°C for 2 min, 35 cycles at 94°C for 15 s, 60°C for 15 s and 72°C for 15 s and 1 cycle at 72°C for 7 min before holding the reactions at 4°C.

Samples were tested for IHNV and VHSV using nested RT-PCR with primers and conditions described by Batts and Winton [42] and [43], respectively, with minor modifications. The RT-PCR was performed using 50 μl reaction mixture utilizing EzrTth RT-PCR kit (Applied Biosystems) as follows: EzrTth DNA Polymerase-2.5 units, forward primer-0.4 uM, reverse primer -0.4 uM, Mn-2.5 mM, dNTPs -200 uM each, 5× PCR buffer-10 μl, ddH_2_O-19 μl and 5 μl RNA template. For IHNV, the primers consisted of IHNV 1F primer and IHNV 1R primer whereas for VHSV they were VHSV 1F primer and VHSV 1R primer. The amplification was performed in Perkin Elmer Gene Amp PCR System 2400 using the following conditions: 1 cycle at 50°C for 15 min, 1 cycle at 95°C for 2 min, 25 cycles at 95°C for 30 s, 50°C for 30 s and 72°C for 60 s, 1 cycle at 72°C for 1 min and 1 cycle at 72°C for 7 min before soaking at 4°C. The second amplification was done using AmpliTaq Gold (Applied Biosystems) utilizing 50 μl mixture as follows: AmpliTaq Gold Polymerase-2.5 units, IHNV 2F primer – 0.4 uM, IHNV 2R primer-0.4 uM for IHNV or VHSV 2F primer – 0.4 uM, VHSV 2R primer-0.4 uM for VHSV, Mg-2 mM, dNTPs -200 mM each, 10× PCR buffer – 5 μl, ddH_2_O- 33.5 μl and 2 μl RT-PCR mixture. The following cycling conditions were used: 1 cycle of 95°C for 2 min, 25 cycles at 95°C for 30 s, 50°C for 30 s and 72°C for 60 s and 1 cycle at 72°C for 7 min before holding the reactions at 4°C.

For DNA sequencing of ISAV RT-PCR products, total RNA was extracted from 300 μl of tissue homogenate using Trizol LS^® ^(Invitrogen). The RT-PCR was carried out using the Titan^® ^One Tube RT-PCR (Roche Diagnostic), in a PTC-200 DNA Engine Peltier thermal cycler (MJ Research, Inc.) using oligonucleotide primers and cycling conditions as previously described [19,44]. The PCR products were then either directly sequenced or they were cloned into the pCRII vector using a TOPO TA cloning kit (Invitrogen) in preparation for nucleotide sequencing. Plasmid DNA for sequencing was prepared as described before [45]. DNA sequencing was performed as previously described [19] by ACGT Corporation (Toronto, Ontario, Canada). DNA Sequencing was done either directly on RT-PCR products or on plasmid DNA containing the cloned RT-PCR products obtained from reactions using total RNA from tissue samples. Sequence analysis used the BLAST programs [46], the Sequence Manipulation suite version 2 [47], and the FASTA program package for personal computers [48].

## Authors' contributions

MGG made the veterinary investigation of the outbreak, performed the necropsy and histological analysis, coordinated the laboratory investigation, and helped to write the manuscript. AA coordinated the laboratory investigation and performed the immunohistochemistry analysis. MJTK performed the RT-PCR and sequence analysis of RNA segment 5 and edited the manuscript. DBG performed the histological evaluation and edited the manuscript. CVY performed the RT-PCR for ISAV, IHNV and VHSV, provided total RNA of U24636 and U24637 to the OIE Ref Lab, and edited the manuscript. HG performed RT-PCR on tissue homogenates and cell culture lysates. AL and MC isolated the virus in cell culture. FA coordinated all the MH laboratory procedures. MI made the veterinary investigation of the increased mortality in the outbreak. MJ coordinated the MH laboratory investigation and wrote the manuscript. FSBK coordinated the viral analysis and DNA sequence analysis of RNA segment 5, and helped to write the manuscript. All authors read and approved the final manuscript.

## Supplementary Material

Additional File 1 Additional clinical signs in affected Atlantic salmon (*Salmo salar*) from the 2007 infectious salmon anaemia (ISA) outbreak in Chile. A marine-fish cage on the Atlantic salmon grow-out site showing market-size Atlantic salmon affected in the ISA outbreak. Click here for file

Additional File 2 Additional clinical signs in affected Atlantic salmon (*Salmo salar*) from the 2007 infectious salmon anaemia (ISA) outbreak in Chile. Affected Atlantic salmon with exophthalmia from the ISA outbreak. Click here for file

Additional File 3 Additional clinical signs in affected Atlantic salmon (*Salmo salar*) from the 2007 infectious salmon anaemia (ISA) outbreak in Chile. Fluid drawn with a 3-ml syringe from the pericardial sac of affected Atlantic salmon with Hydropericardium from the ISA outbreak. Click here for file

Additional File 4 Additional clinical signs in affected Atlantic salmon (*Salmo salar*) from the 2007 infectious salmon anaemia (ISA) outbreak in Chile. Affected Atlantic salmon with abdominal jaundice from the ISA outbreak. Click here for file

Additional File 5 Additional clinical signs in affected Atlantic salmon (*Salmo salar*) from the 2007 infectious salmon anaemia (ISA) outbreak in Chile. Affected Atlantic salmon with haemorrhages in stomach from the ISA outbreak. Click here for file

Additional File 6 Table 1. The data provided represent percent sequence identities of the viral Fusion (Segment 5) gene of Chilean ISAV and selected isolates of Genotype I (European) and Genotype II (North American). Click here for file

Additional File 7 Table 2. The data provided represent percent sequence identities of the viral Haemagglutinin-Esterase (Segment 6) gene of Chilean ISAV and selected isolates of Genotype I (European) and Genotype II (North American). Click here for file
