# Supplementary figures and images for: First detection, isolation and molecular characterization of infectious salmon anaemia virus associated with clinical disease in farmed Atlantic salmon (Salmo salar) in Chile
Source: BMC Vet Res. 2008 Aug 4;4:28. doi: 10.1186/1746-6148-4-28 (PMC2519066; doi:10.1186/1746-6148-4-28)

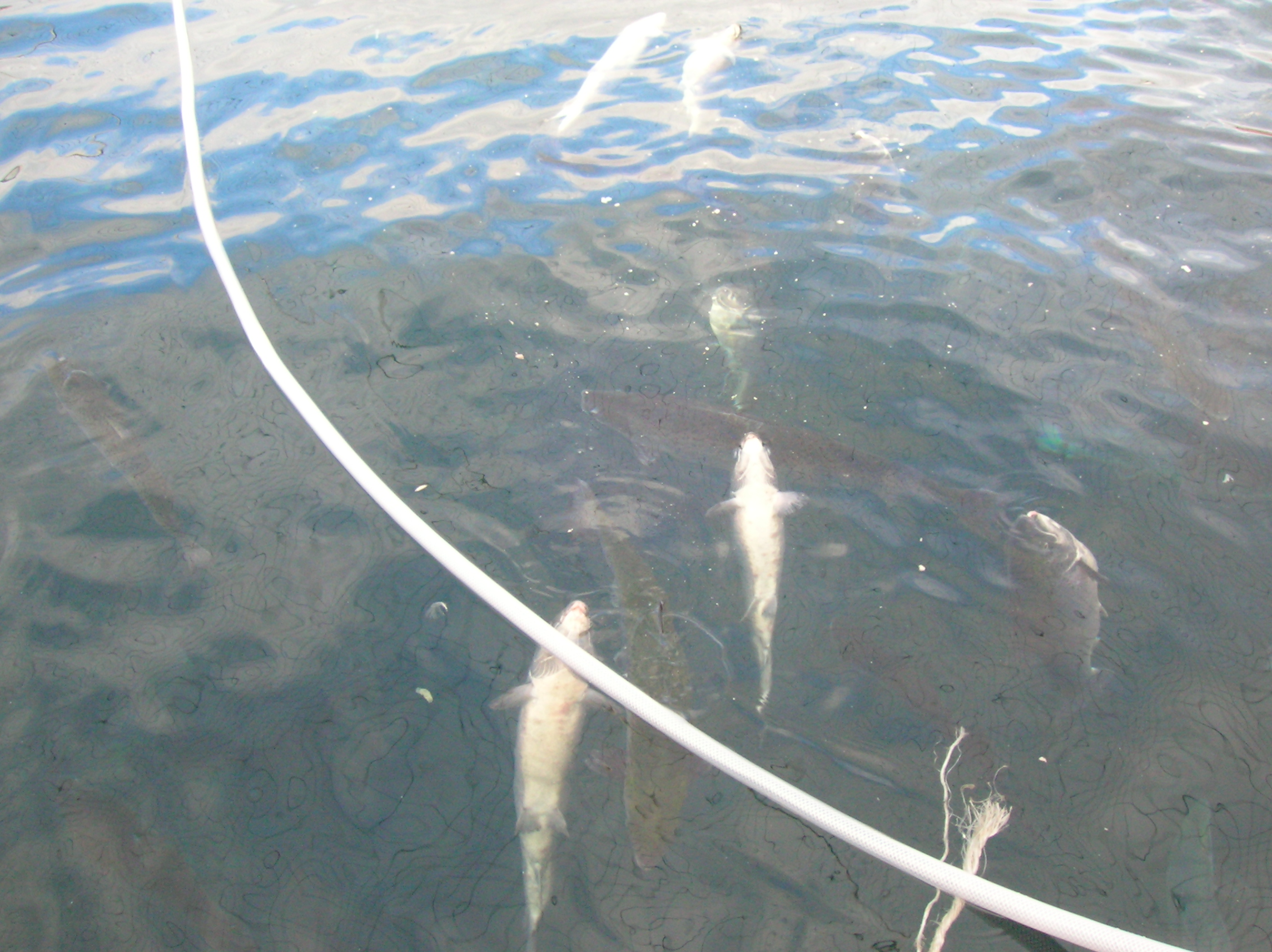

Supplement: Additional File 1 — Additional clinical signs in affected Atlantic salmon (Salmo salar) from the 2007 infectious salmon anaemia (ISA) outbreak in Chile. A marine-fish cage on the Atlantic salmon grow-out site showing market-size Atlantic salmon affected in the ISA outbreak. [file 1746-6148-4-28-S1.doc]

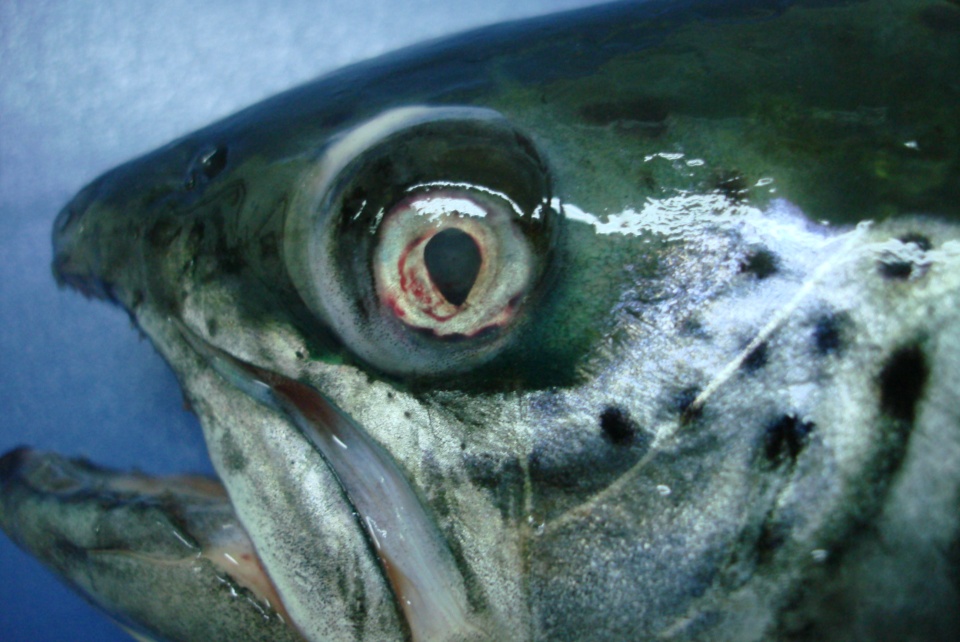

Supplement: Additional File 2 — Additional clinical signs in affected Atlantic salmon (Salmo salar) from the 2007 infectious salmon anaemia (ISA) outbreak in Chile. Affected Atlantic salmon with exophthalmia from the ISA outbreak. [file 1746-6148-4-28-S2.doc]

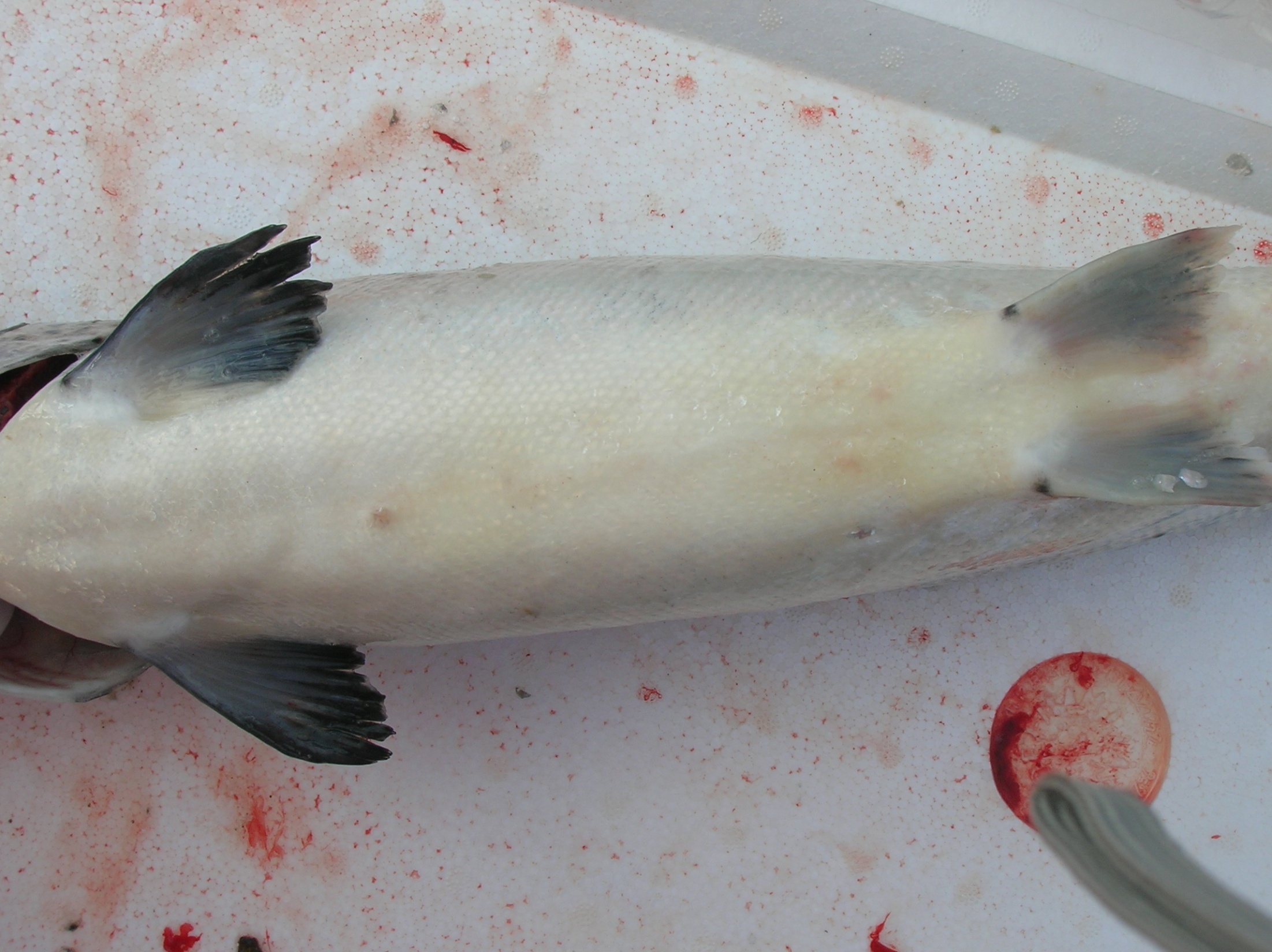

Supplement: Additional File 4 — Additional clinical signs in affected Atlantic salmon (Salmo salar) from the 2007 infectious salmon anaemia (ISA) outbreak in Chile. Affected Atlantic salmon with abdominal jaundice from the ISA outbreak. [file 1746-6148-4-28-S4.doc]

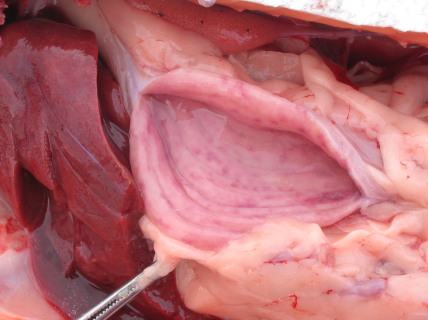

Supplement: Additional File 5 — Additional clinical signs in affected Atlantic salmon (Salmo salar) from the 2007 infectious salmon anaemia (ISA) outbreak in Chile. Affected Atlantic salmon with haemorrhages in stomach from the ISA outbreak. [file 1746-6148-4-28-S5.doc]
