# Supplementary material for: First detection, isolation and molecular characterization of infectious salmon anaemia virus associated with clinical disease in farmed Atlantic salmon (Salmo salar) in Chile
Source: BMC Vet Res. 2008 Aug 4;4:28. doi: 10.1186/1746-6148-4-28 (PMC2519066; doi:10.1186/1746-6148-4-28)
Supplement: Additional File 6 — Table 1. The data provided represent percent sequence identities of the viral Fusion (Segment 5) gene of Chilean ISAV and selected isolates of Genotype I (European) and Genotype II (North American). [file 1746-6148-4-28-S6.doc]

**Table 1.** Pairwise sequence comparison of the viral Fusion (Segment 5) gene of Chilean ISAV and selected isolates of Genotype I (European) and Genotype II (North American)^1^

| ISAV isolate | U24636 | U24637 | 24909 | SK779/06 | 390/98 | 810/9/99 | 04-085-1 | NBISA01 | 98-280-2 | 7833-1 |
| --- | --- | --- | --- | --- | --- | --- | --- | --- | --- | --- |
| U24636^2^ (Chile 2007) | **-** | **98.6** | **99.9** | **98.3** | **98.2** | **98.1** | **97.9** | 74.7 | 74.6 | 74.6 |
| U24637 (nt 45-337) (Chile 2007) | **nd**^5^ | **-** | **98.6** | **96.6** | **96.6** | **96.6** | **95.9** | 72.5 | 72.7 | 72.2 |
| 24909 (nt 1-788) (Chile 2007) | **nd** | **96.9** | **-** | **98.7** | **98.2** | **98.1** | **98.0** | 74.5 | 74.4 | 74.4 |
| SK779/06 (European HPR0)^3^ | **97.1** | **nd** | **nd** | **-** | **98.4** | **98.1** | **98.3** | 76.2 | 76.0 | 76.0 |
| Scotland 390/98 | **96.3** | **nd** | **nd** | **99.1** | **-** | **99.0** | **98.7** | 76.3 | 76.1 | 76.1 |
| Norway 810/9/99 | **95.6** | **nd** | **nd** | **98.4** | **99.3** | **-** | **98.3** | 76.1 | 76.0 | 76.0 |
| RPC/NB-04-085-1 | **96.0** | **nd** | **nd** | **98.6** | **99.3** | **98.6** | **-** | 76.5 | 76.4 | 76.4 |
| NBISA01 | 82.8 | nd | nd | 84.5 | 84.5 | 84.0 | 84.2 | - | 99.3 | 99.9 |
| RPC/NB-98-280-2 | 81.8 | nd | nd | 84.0 | 84.0 | 83.6 | 83.8 | 98.4 | - | 99.3 |
| 7833-1 (Chile 1999)^4^ | 81.8 | nd | nd | 84.0 | 84.0 | 83.6 | 83.8 | 99.5 | 98.4 | - |

^1^Values above the diagonal are nucleotide sequence identities (%); values below the diagonal are deduced amino acid sequence identities (%). Bold text denotes sequence identities among Genotype I ISAV isolates

^2^ISAV U24636 (Chile 2007) segment 5 sequence is the full-length open reading frame for the Fusion protein; only partial sequences were determined for ISAV U24637 (nucleotide positions 45-337) and Biovac ID 24909 (nucleotide positions 1-788). The 11-amino acid insert is in the 3’ part of the gene and therefore not sequenced in U24637 and 24909. The Chile 2007 sequences are available in GenBank, accession #s EU130923 for U24636; EU486161 for U24637; and EU486160 for 24909. An alignment in the in the proteolytic cleavage site of the F protein is shown in Figure 5.

^3^ISAV SK779/06 (which is European HPR0 virus) was detected in 2006 by RT-PCR of tissues from Atlantic salmon with gill disease [20].

^4^ISAV 7833-1 (Chile 1999) was isolated from Coho salmon in 1999, and is of North American genotype [12].

^5^nd denotes not done (because it is partial sequence).
