# Supplementary material for: First detection, isolation and molecular characterization of infectious salmon anaemia virus associated with clinical disease in farmed Atlantic salmon (Salmo salar) in Chile
Source: BMC Vet Res. 2008 Aug 4;4:28. doi: 10.1186/1746-6148-4-28 (PMC2519066; doi:10.1186/1746-6148-4-28)
Supplement: Additional File 7 — Table 2. The data provided represent percent sequence identities of the viral Haemagglutinin-Esterase (Segment 6) gene of Chilean ISAV and selected isolates of Genotype I (European) and Genotype II (North American). [file 1746-6148-4-28-S7.doc]

**Table 2.** Pairwise sequence comparison of the viral Haemagglutinin-Esterase (Segment 6) gene of Chilean ISAV and selected isolates of Genotype I (European) and Genotype II (North American)^1^

| ISAV isolate | 1065 | SK779/06 | 390/98 | 810/9/99 | 04-085-1 | NBISA01 | 98-280-2 | 7833-1 |
| --- | --- | --- | --- | --- | --- | --- | --- | --- |
| 1065^2^ (Chile 2007) | **-** | **98.6** | **99.5** | **99.1** | **95.7** | 79.4 | 79.4 | 79.3 |
| SK779/06 (European HPR0)^3^ | **98.5** | **-** | **98.6** | **98.3** | **96.2** | 78.6 | 78.8 | 78.0 |
| Scotland 390/98 | **98.9** | **98.2** | **-** | **99.5** | **95.7** | 79.1 | 79.2 | 79.0 |
| Norway 810/9/99 | **98.4** | **97.4** | **99.0** | **-** | **95.4** | 78.7 | 78.8 | 78.5 |
| RPC/NB-04-085-1 | **93.1** | **94.4** | **92.8** | **91.9** | **-** | 77.8 | 78.2 | 77.7 |
| NBISA01 | 84.3 | 84.6 | 84.3 | 84.0 | 83.3 | - | 98.6 | 98.9 |
| RPC/NB-98-280-2 | 84.4 | 82.2 | 84.4 | 84.1 | 80.7 | 93.5 | - | 98.9 |
| 7833-1 (Chile 1999)^4^ | 83.7 | 84.0 | 82.8 | 83.4 | 82.7 | 97.1 | 94.1 | - |

^1^ Values above the diagonal are nucleotide sequence identities (%); values below the diagonal are deduced amino acid sequence identities (%). Bold text denotes sequence identities among Genotype I ISAV isolates.

^2^ISAV 1065 (Chile 2007) segment 6 sequence is the partial sequence determined by Norwegian Veterinary Institute and is available in GenBank, accession # AM941715. An alignment in the HPR is shown in Figure 6.

^3^ISAV SK779/06 (which is European HPR0 virus) was detected in 2006 by RT-PCR of tissues from Atlantic salmon with gill disease [20].

^4^ISAV 7833-1 (Chile 1999) was isolated from Coho salmon in 1999, and is of North American genotype [12].
