# Supplementary material for: First detection, isolation and molecular characterization of infectious salmon anaemia virus associated with clinical disease in farmed Atlantic salmon (Salmo salar) in Chile
Source: BMC Vet Res. 2008 Aug 4;4:28. doi: 10.1186/1746-6148-4-28 (PMC2519066; doi:10.1186/1746-6148-4-28)

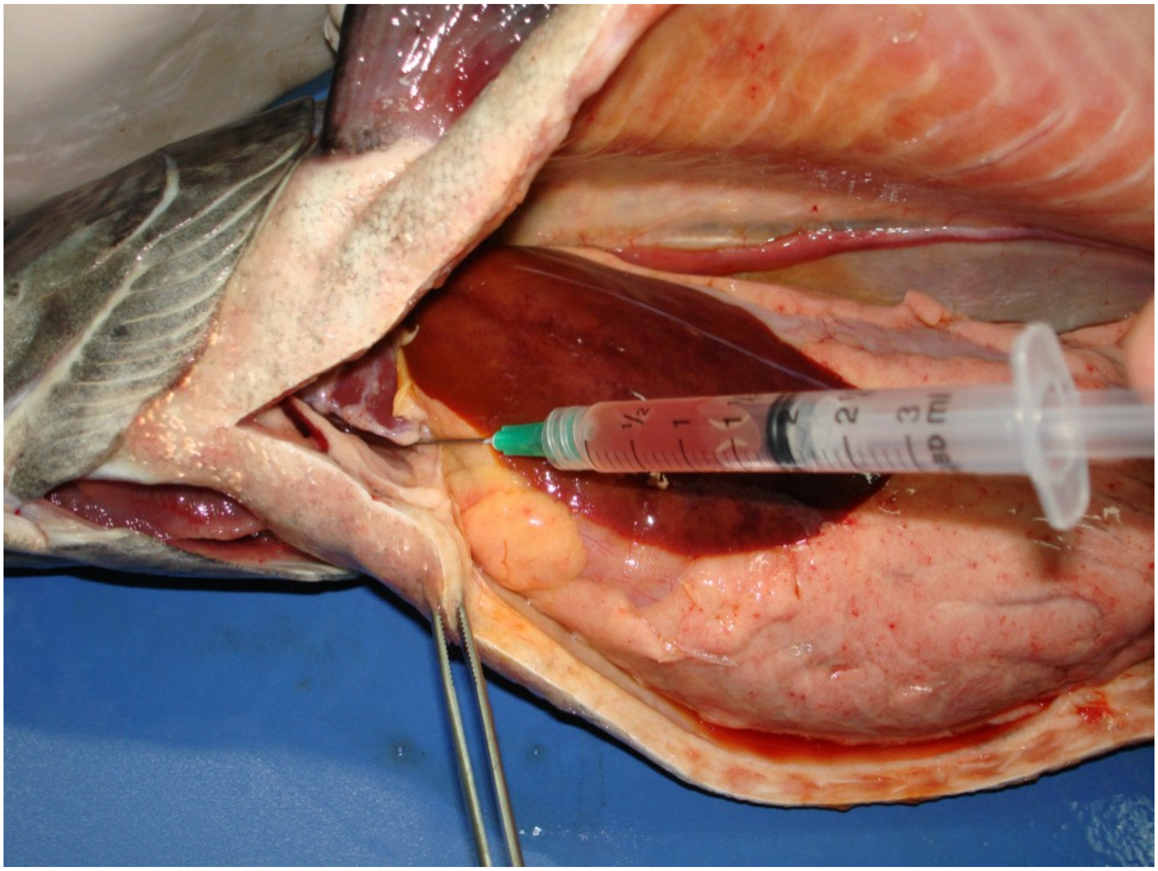

Supplement: Supplementary file 4 [file 1746-6148-4-28-S3.doc]
